# Antioxidant action of SMe1EC2, the low-basicity derivative of the pyridoindole stobadine, in cell free chemical models and at cellular level

**DOI:** 10.2478/intox-2014-0005

**Published:** 2014-07-16

**Authors:** Aneta Balcerczyk, Grzegorz Bartosz, Joanna Drzewinska, Łukasz Piotrowski, Łukasz Pulaski, Milan Stefek

**Affiliations:** 1 Department of Molecular Biophysics, University of Lodz, Lodz, Poland; 2 Department of Biochemistry and Cell Biology, University of Rzeszow, Rzeszow, Poland; 3 Laboratory of Transcriptional Regulation, Center of Medical Biology, PAS, Lodz, Poland; 4 Institute of Experimental Pharmacology and Toxicology, Slovak Academy of Sciences, Bratislava, Slovakia

**Keywords:** antioxidant, hexahydropyridoindole, SMe1EC2, stobadine, oxidative stress

## Abstract

The aim of the study was to evaluate the antioxidant action of SMe1EC2, the structural analogue of the hexahydropyridoindole antioxidant stobadine. The antiradical activity of SMe1EC2 was found to be higher when compared to stobadine, as determined both in cell-free model systems of AAPH-induced oxidation of dihydrorhodamine 123 and 2′,7′-dichloro-dihydrofluorescein diacetate, and in the cellular system of stimulated macrophages RAW264.7. Analysis of proliferation of HUVEC and HUVEC-ST cells revealed absence of cytotoxic effect of SMe1EC2 at concentrations below 100 µM. The antioxidant activity of SMe1EC2, superior to the parent drug stobadine, is accounted for by both the higher intrinsic free radical scavenging action and by the better bioavailability of the low-basicity SMe1EC2 relative to the high-basicity stobadine.

## Introduction

Considering the antioxidant stobadine as a lead (Horakova and Stolc 1998), a number of structurally related hexahydropyridoindole congeners have been designed, synthesized and characterized (Juranek *et al.*, 2010; Rackova *et al.*, 2006, Stolc *et al.*, 2008). Modification of the stobadine molecule by aromatic electron donating substitution was reported to enhance the intrinsic free radical scavenging activity, while variations of the N2 substituent provided a synthetically accessible way to modulate the biological availability by affecting both lipophilicity and basicity of the molecule, without changing significantly the free radical scavenging activity (Rackova *et al.*, 2002; Rackova *et al.*, 2006)

**Figure 1:**
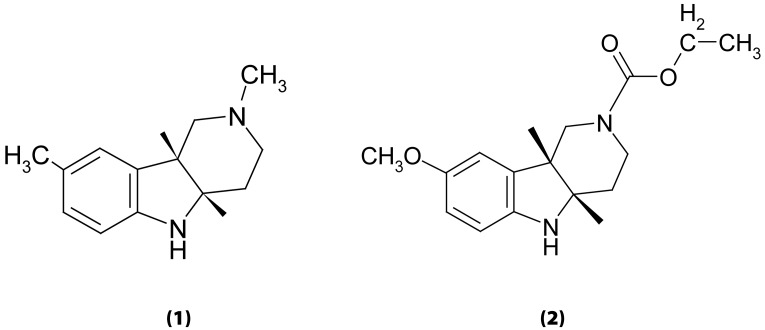
Chemical structure of stobadine, [(–)-*cis*-2,8-dimethyl-2,3,4,4a,5,9b-hexahydro-1H-pyrido[4,3b]indole, (**1**)] and SMe1EC2 [(±)-*cis*-8-methoxy-1,3,4,4a,5,9b-hexahydro-pyrido[4,3-b]indole-2-carboxylic acid ethyl ester, (**2**)].

The subject of the present study was SMe1EC2, the 8-methoxy analogue of stobadine with an acyl substituent at the position N2 (Figure 1). In extensive preclinical studies, SMe1EC2 revealed significant neuroprotection in the murine model of acute head trauma (Stolc *et al.*, 2006, 2008) and in *in vitro* rat hippocampal slices exposed to transient hypoxia/reoxygenation (Gasparova *et al.*, 2009, 2010, 2011, 2014). Under conditions of experimental diabetes of rats, SMe1EC2 attenuated endothelial injury and restored the reduced endothelium-mediated relaxation in diabetic animals (Sotnikova *et al.*, 2011). SMe1EC2 improved the viability of HT22 neuronal cells in culture exposed to high glucose and attenuated indices of oxidative stress (Rackova *et al.*, 2009). The compound protected efficiently rat pancreatic INS-1E β cell cultures against cytotoxic effects of hydrogen peroxide (Rackova *et al.*, 2011). Preclinical toxicology tests revealed a remarkably low acute toxicity of SMe1EC2 in mice, regardless the way of administration (Stolc *et al.*, 2010). Contrary to stobadine, SMe1EC2 did no possess any α-adrenolytic action (Stolc *et al.*, 2010). In a prenatal developmental toxicity study in rats, SMe1EC2 exerted neither embryotoxic nor teratogenic effects on rat fetuses and their postnatal development, nor were any signs of maternal toxicity found (Ujhazy *et al.*, 2008, 2011).

In our previous study (Stefek *et al.*, 2013), on applying a DPPH test, we reported efficient free radical scavenging activity of SMe1EC2, comparable with that of the standard trolox. In the cellular system of isolated erythrocytes, SMe1EC2 protected red blood cells against free-radical-initiated hemolysis. The overall antioxidant efficacy of SMe1EC2, relative to the reference antioxidant stobadine, was strongly affected by the lipophilicity of the initiating free radical species. In the system of t-BuOOH/isolated erythrocytes, a model cellular system of endogenously generated peroxyl radicals, SMe1EC2 significantly exceeded the parent stobadine in its antioxidant action.

The first part of the present study reports the evaluation of the intrinsic antiradical activity of SMe1EC2 in comparison with stobadine in the cell-free model system of 2,2‘-azobis(2-amidinopropane) hydrochloride (AAPH)-induced oxidation of dihydrorhodamine 123 (H_2_R123) and 2′,7′-dichloro-dihydrofluorescein diacetate (H_2_DCF DA). Further the overall antioxidant action of the compound was studied in the cellular system of mouse macrophages RAW 264.7. Finally, cytotoxicity of SMe1EC2 was examined in primary human umbilical vein endothelial cells (HUVEC) and immortalized human umbilical vein endothelial cells (HUVEC-ST).

## Materials and methods

### Chemicals

SMe1EC2 and stobadine (Figure 1) were synthesized at the Institute of Experimental Pharmacology and Toxicology, Slovak Academy of Sciences, and were available as hydrochlorides. AAPH was obtained from FLUKA Chemie GmbH (Steinheim, Germany). Cell culture reagents: fetal bovine serum, medium M199, Dulbecco's modified Eagle's medium (DMEM), collagenase II and antibiotics were purchased from Invitrogen (Paisley, UK). Epidermal growth factor (EGF) was supplied by Becton Dickinson (San Jose, CA, USA). Fluorescent probes, H_2_R123 and H_2_DCF DA, were obtained from Molecular Probes, Invitrogen Corporation (Leiden, The Netherlands). Other chemicals were purchased from local commercial sources and were of analytical grade quality.

### Cell culture

HUVEC were isolated from veins of freshly collected umbilical cords, by collagenase type II digestion (Jaffe *et al.,* 1973) and used for the experiments at passage 2–4. They were cultured in medium 199 containing 20% heat-inactivated fetal bovine serum, 10 U/ml penicillin, 50 µg/ml streptomycin, 5 µg/ml sodium heparin and 10 ng/ml epidermal growth factor (EGF). The cells were grown at 37 °C and 5% CO_2_ on plastic flasks coated with 1% gelatin. The cultured cells showed the typical cobblestone-like appearance, and were identified as endothelial cells by fluorescence activated cell sorting (FACS) analysis for von Willebrand factor with ECA-4 antibodies (kindly donated by Dr. Monica Spadofora-Ferreira, University of Sao Paulo, Brazil; Spadafora-Ferreira *et al.*, 2000). SW620 colorectal adenocarcinoma cells were used as a negative control.

HUVEC-ST were cultured in OptiMEM medium supplemented with 3.5% heat-inactivated fetal bovine serum and antibiotics (10 U/ml penicillin, 50 µg/ml streptomycin). The HUVEC-ST cell line was obtained from Prof. C. Kieda (Orleans, France).

Mouse RAW 264.7 macrophages, kindly donated by Dr. Pawel Lipinski (Institute of Genetics and Animal Breeding, Jastrzebiec, Poland), were cultured in DMEM containing 10% fetal bovine serum heat-inactivated and 50 µg/ml gentamycin.

### MTT assay

Proliferation of HUVEC/HUVEC-ST was assessed by measuring the ability of live cells to metabolize MTT, 3-(4,5-dimethylthiazol-2-yl)-2,5-diphenyltetrazolium bromide, to formazan. Cells were seeded onto gelatin-coated 96-well plates at a density of 3×10^3^ cells per well (HUVEC) or 1×10^3^ cells per well (HUVEC-ST). After overnight culture, the cells were treated for 72 h by stobadine and its derivatives in a concentration range of 5–200 µM. At the end of treatment, the cell monolayers were rinsed with HBSS and fresh medium containing MTT (final MTT concentration of 333 mg/ml) was added. After 3 h the medium was removed and formazan crystals were dissolved in DMSO. Absorbance was read at 590 nm.

### Migration/Wound-healing Assay

Migration of cells was tracked using the Olympus automated phase-contrast microscope image analysis system. Scratch on confluent HUVEC monolayer was performed using the migration inserts (Ibidi^®^, Germany). Cells were washed twice with EBM-2 serum-free medium to remove detached and damaged cells and fresh complete growth medium was added. To exclude the influence of proliferation on wound closure, the medium was supplemented with an inhibitor of cell proliferation, mitomycin C (10 µg/ml) (Sigma). Wound size was measured a) immediately, b) 6h, and c) 12h after removing the insert. Estimation of ECs migration was performed using Metamorph software.

Migration of cells (% of recovery) was quantified by using the equation:

% R = [1 – (wound area at T_t_/wound area at T_0_] × 100%

where:

T_t_ - wound area at indicated time after the injury

T_0_ - wound area immediately after the injury (0 h)

## Results

### Cell-free model

**Figure 2:**
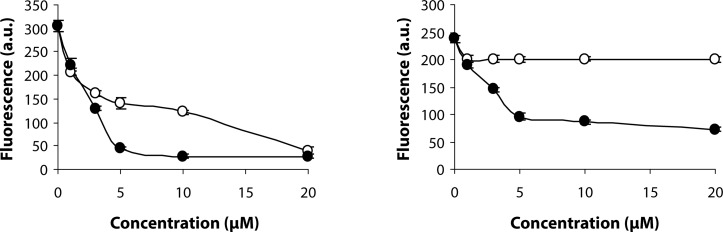
SMe1EC2 (-●-) and stobadine (-○-) protect H_2_R123 (a) and H_2_DCF DA (b) from AAPH induced oxidation in a cell-free system. Results are presented as means ± SD from at least three measurements.

As shown in Figure 2, SMe1EC2 was found more efficient than stobadine in protecting H_2_R123 and H_2_DCF DA from AAPH-induced oxidation in a cell-free system.

### Cellular systems

#### Studies in stimulated macrophages RAW 264.7

**Figure 3:**
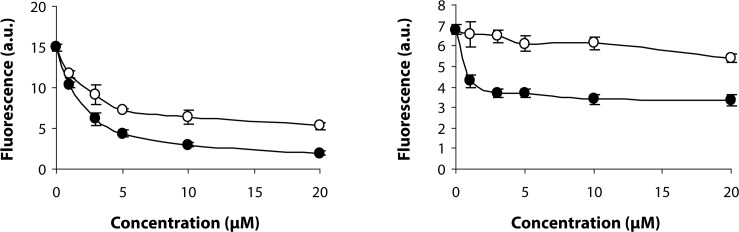
Effect of SMe1EC2 (-●-) and stobadine (-○-) on oxidation of H_2_R123 (a) or H_2_DCF DA (b) by RAW 264.7 macrophages. RAW 264.7 macrophages were stimulated with 100 µg/ml of LPS for 16 h and with 100 nM PMA for 30 min in a complete medium.

Determination of reactive oxygen/nitrogen species production by using fluorogenic probes confirmed the antioxidant properties of the compounds analyzed. Macrophages RAW 264.7 were used as a model. Nitric oxide production was stimulated by 16-h treatment of the cells with 100 ng/ml of lipopolysaccharide (LPS). Analysis of reactive oxygen species production was performed in cells stimulated with LPS (100 ng/ml, 16 h) and phorbol ester (PMA; 100 nM, 30 min), by using H_2_R123. Both compounds tested inhibited ROS/RNS production. SMe1EC2 revealed stronger antioxidant properties than stobadine (Figure 3). Based on H_2_DCF DA oxidation (Ischiropoulos *et al.*, 1999), stobadine in the range of concentrations of 1–20 µM decreased the nitric oxide production by about 15%. The effect of SMe1EC2 was significantly stronger (inhibition was up to 50%).

#### Proliferation of HUVEC and HUVEC-ST

**Figure 4:**
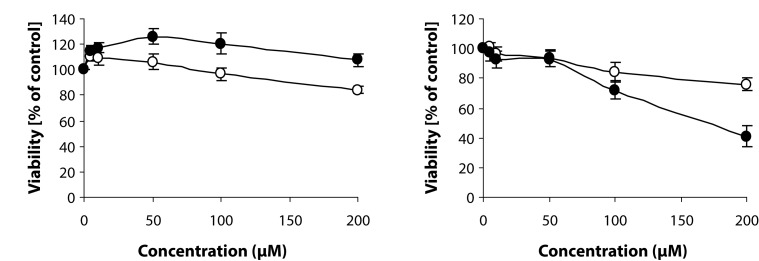
Effect of SMe1EC2 (-●-) and stobadine (-○-) on proliferation of HUVEC (a) and HUVEC-ST (b). MTT assay after 72-h incubation of cells with the compounds.

Analysis of proliferation under 72-h treatment of cells with stobadine and its derivative showed (Figure 4) that, in the range of concentrations 5–200 µM, SMe1EC2 slightly stimulated proliferation of HUVEC (Figure 4a, up to 120% of control), but only of primary and not of immortalized endothelial cells (HUVEC-ST), while stobadine had no significant effect on cell growth. In the case of HUVEC-ST, the cytotoxic effect of SMe1EC2 and stobadine was recorded at concentrations ≥ 100 µM (Figure 4b).

#### Migration of HUVECs by using wound-healing assay

**Figure 5:**
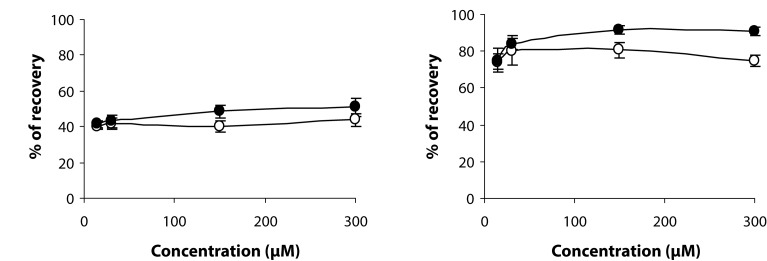
Effect of SMe1EC2 (-●-) and stobadine (-○-) on the migration of HUVEC after 12 (a) or 24 (b) hours.

Migration of HUVECs, by using wound-healing technique, was analyzed after 12 h and 24 h. The percentage of recovery was slightly increased in the case of SMe1EC2-treated cells. Stobadine did not exert any statistically significant effect (Figure 5).

## Discussion

The pyridoindole stobadine has been postulated as a chain-breaking antioxidant characterized by the ability to scavenge chain-propagating peroxyl radicals (Steenken *et al.*, 1992; Stefek *et al.*, 1992; Kagan *et al.*, 1993; Stefek and Trnkova 1996). The center of the antioxidant activity of stobadine and related substituted pyridoindoles was identified to reside at the indolic nitrogen (Rackova *et al.*, 2002). Structural alterations in the close proximity of the indolic nitrogen, especially aromatic substitution in positions *o* and *p*, were found to influence the antioxidant efficacy (Rackova *et al.*, 2002, Rackova *et al.*, 2006). Moreover, alteration in the synthetically accessible position N2 provides the opportunity to vary basicity and lipophilicity of the compounds, thus optimizing bioavailability without affecting the intrinsic antiradical activity (Rackova *et al.*, 2006).

The subject of the present study was SMe1EC2, the methoxy analogue of stobadine, whose acyl substituent at the position N2 was expected to decrease the basicity of this site without changing significantly the lipohilicity of the molecule. At the same time, the electron donating methoxy group at the aromatic position 8 was supposed to contribute to the elevation of the free radical scavenging activity compared to the parent stobadine.

In the first series of experiments, the intrinsic antioxidant activity of SMe1EC2 in comparison to stobadine was tested in a cell-free system comprising oxidant-sensitive fluorescent probes, H_2_R123 and H_2_DCF (Crow 1997; Kalyanaraman *et al.*, 2012), exposed to AAPH-derived peroxyl radicals. Under the experimental conditions used, SMe1EC2 protected more efficiently H_2_R123 and H_2_DCF from their oxidation than did stobadine.

In homogeneous cell-free systems, antioxidant activity stems from an intrinsic chemical reactivity towards radicals. In membranes, however, the relative reactivities may be different since they are determined also by additional factors such as location of the antioxidant and radicals, ruled predominantly by their actual distribution ratios between water and lipid compartments. As we reported earlier (Stefek *et al.*, 2013), SMe1EC2 and stobadine have similar lipophilicities, characterized by the corresponding log P values of 1.95 and 1.79, respectively. Yet their actual distribution ratios at pH 7.4 were shown to differ profoundly (calculated log D_SMe1EC2_=1.78 vs. calculated log D_stobadine_=–0.05) as a result of basicity variance of SMe1EC2 vs. stobadine, characterized by respective pKa values, –3.7 vs. 8.5.

SMe1EC2 was found more effective than stobadine in scavenging reactive oxygen/nitrogen species in stimulated macrophage RAW 264.7 cell cultures by using H_2_R123 and H_2_DCF DA as fluorogenic probes.

Studies in HUVEC cell line revealed that both SMe1EC2 and stobadine, in concentrations up to 200 µM, did not decrease significantly the viability of the cells. On the other hand, SMe1EC2 slightly stimulated HUVEC proliferation at 50 µM concentration. Yet, in relation to HUVEC-ST, some cytotoxic effect of both compounds studied was recorded at concentrations ≥100 µM. Moreover, SMe1EC2 slightly stimulated the migration of HUVEC while stobadine failed to exert any effect.

To conclude, the present outcomes, in the context of preceding findings, indicate that modification of the hexahydropyridoindole skeleton of stobadine may yield congeners with increased antiradical efficacy and bioavailability, and that at reduced side effects.
